# Short Hydration Regimen in Cisplatin-Based Chemotherapy and Its Impact on Nephrotoxicity: A Unicentric Prospective Study

**DOI:** 10.7759/cureus.79774

**Published:** 2025-02-27

**Authors:** Ana Raquel Teixeira, Diana Mata, Hugo Ferreira, Ana Paiva, Maria J Pelayo, Carla Rafael, Joaquina Maurício, Rita Calisto, Maria Cassiano Neves

**Affiliations:** 1 Medical Oncology, Instituto Português Oncologia do Porto Francisco Gentil, Porto, PRT; 2 Nephrology, Instituto Português Oncologia do Porto Francisco Gentil, Porto, PRT; 3 Pharmaceutics, Instituto Português Oncologia do Porto Francisco Gentil, Porto, PRT; 4 Nursing, Instituto Português Oncologia do Porto Francisco Gentil, Porto, PRT; 5 Epidemiology, Instituto Português Oncologia do Porto Francisco Gentil, Porto, PRT

**Keywords:** acute kidney injury, cancer chemotherapy protocol, chemotherapy, cisplatin, drug toxicity, outpatient care

## Abstract

Nephrotoxicity is common when cisplatin is used and hydration during treatment is nephroprotective, but the optimal volume, composition, and duration are unknown. We reviewed our institutional intravenous hydration regimen (total 2500 mL in 5 hours versus previously used 3000 mL in 7 hours) in cisplatin >50 mg/m^2^ schemes and we aimed to confirm the safety of this change by prospectively evaluating the incidence of acute kidney injury (AKI). We included 105 patients in our cohort. However, for comparison, we only considered 96 cases and 191 controls (ratio 2:1), due to the impossibility of matching all cases. The proportion of patients without Kidney Disease Improving Global Outcomes (KDIGO) AKI stage ≥2 (serum creatinine ≥2.0-2.9 times baseline) was 100.0% (n=96) after the first treatment and 97.8% (n=88) after the second treatment in our cohort. There was no difference when compared with the historical cohort of patients who received the previous cisplatin hydration regimen (p=1 for the first treatment and p=0.92 for the second treatment). AKI was the reason for cisplatin discontinuation in three patients (2.9%). Our results support the safety of a shorter and lower volume intravenous hydration during cisplatin treatment, which is more comfortable for patients and allows a better health resource allocation.

## Introduction

Cisplatin is an anti-neoplastic drug commonly used in a variety of solid tumors, such as head and neck, esophagus, bladder, ovarian, lung, and bladder cancers [1,2]. One of the most important toxicities is cisplatin-induced nephrotoxicity (CIN), with a direct impact on dosing and/or dose intensity and eventually leading to the discontinuation of effective cancer treatments [2,3].

CIN affects around one-third of treated patients and the most common presentation is acute kidney injury (AKI) [2,4,5]. Cisplatin is mainly excreted in the urine in the 24 hours following cisplatin administration and AKI usually occurs 10 days after cisplatin administration [1,6,7].

Cisplatin-induced AKI (cis-AKI) is frequently reversible, with a recovery period of 2-4 weeks, but in some cases may progress to chronic damage [2,4]. The CIN mechanisms are complex [4]. Cisplatin accumulates in renal parenchymal cells, especially in proximal tubules, which leads to cell damage and death [4-6]. In addition, there is stimulation of an immune response that exacerbates local damage and cisplatin appears to damage the renal vasculature [6,7].

The risk factors for CIN may be divided into patient- and treatment-related. Patient-related risk factors include sociodemographic characteristics (older age, female sex), comorbidities (smoking, arterial hypertension, diabetes, ischemic heart disease, pre-existing renal insufficiency), existing drug therapies or regimens (diuretics, renin-angiotensin system inhibitors, non-steroidal anti-inflammatory drugs, aminoglycosides, paclitaxel) and biological data (hypoalbuminemia, hypomagnesemia) [3,4,8-10]. Treatment-related risk factors are dose, frequency, and cumulative dose [3,4].

The lack of strong evidence regarding the optimal hydration regimen leads to the existence of a wide variety of hydration regimens among health institutions [5,11]. Therefore, we decided to review our center’s cisplatin hydration regimen in order to reduce total treatment time, increase patient comfort, and allow better management in the day hospital. By conducting this prospective study, we aimed to confirm the renal safety of changing the hydration of cisplatin.

## Materials and methods

Study population

This study is prospective and unicentric with a historical control group. The matching criteria for the control group were sex, age, and cancer location. The inclusion criteria were age ≥18 years, diagnostic of solid cancer, treatment with cisplatin >50 mg/m^2^, and creatinine clearance (CrCl) ≥60 mL/min according to the Cockcroft-Gault equation previous to cisplatin treatment. Cisplatin could be administered in monotherapy or in combination with other drugs, with or without concomitant radiotherapy, and in any line of treatment. The exclusion criterion was previous treatment with cisplatin.

The historical control group was planned to have a ratio of 2:1. However, due to the impossibility of matching all cases, we only considered 96 cases and 191 controls for the comparison. The null hypothesis of the proportion of patients without Kidney Disease Improving Global Outcomes (KDIGO) AKI stage ≥2 was 90% and it was based on a previous study with a hydration volume of 3-4 liters and mannitol supplementation in which the incidence of KDIGO AKI stage ≥2 varied between 2.1% and 13% [12]. Using the one-arm binomial method to achieve α of 5% and power of 90%, and assuming a dropout rate of 5%, the sample size required was 105 participants.

Treatment and data collection

Our multidisciplinary team revised the institutional hydration regimen for cisplatin administration according to scientific articles and other centers’ experience. The standard and revised hydration regimens are summarized in Table 1.

**Table 1:** Standard and revised hydration regimens. *Magnesium sulfate 20 mEq if hypomagnesemia (serum magnesium <0.73 mmol/L) D5S: dextrose 5% in sodium chloride 0.9%; NS: normal saline (sodium chloride 0.9%)

Variables	Previous hydration regimen	Revised hydration regimen
Pre-cisplatin	NS solution 1000 mL in 2.5 hours	NS solution 1000 mL in 2 hours
	Potassium chloride 20 mEq	Potassium chloride 10 mEq
	Magnesium sulfate 10 mEq	Magnesium sulfate 10 mEq
Cisplatin dilution	NS solution 1000 mL in 2 hours	NS solution 1000 mL in 2 hours
Post-cisplatin	D5S solution 1000 mL in 2.5 hours	NS solution 500 mL in 1 hour
	Potassium chloride 20 mEq	Magnesium sulfate 10 mEq*
	Magnesium sulfate 10 mEq	Indication for at-home oral hydration
	-	D1 1500 mL, D2-3 2000 mL
Forced diuresis	Mannitol 20% 250 mL	Mannitol 20% 250 mL, if cisplatin dose ≥100 mg/m^2^
	-	Furosemide 20 mg intravenous, if mannitol absent and weight gain ≥3 kg
Total volume	3000 mL	2500 mL
Total time	7 hours	5 hours

Our previous hydration regimen (standard hydration regimen) included pre-cisplatin hydration with 1000 mL in 2.5 hours and post-cisplatin hydration with 1000 mL in 2.5 hours. Cisplatin was also diluted in 1000 mL and infused in 2 hours. All patients received mannitol 20% 250 mL.

The revised hydration regimen included pre-cisplatin hydration with 1000 mL associated in 2 hours and post-cisplatin hydration with 500 mL in 1 hour. An additional dose of magnesium sulfate 10 mEq may be administered in the presence of hypomagnesemia (<0.73 mmol/L). Cisplatin was diluted in 1000 mL and infused in 2 hours. Patients received the indication to drink at least 2000 mL of water on days 2 and 3. Based on the work of Crona et al., who concluded that mannitol should only be administered when the dose of cisplatin is ≥100 mg/m^2^, we changed the administration of mannitol exclusively for this group of patients [5]. In the absence of mannitol administration and final weight gain ≥3 kg, furosemide 20 mg intravenous was administered.

The baseline CrCl was evaluated on day 1 of every cycle for all the patients, independently of the periodicity of the cycles, using the more recent serum creatinine value (necessarily within the previous 30 days). The subsequent CrCl was evaluated with serum creatinine value assessed within 72 hours prior to treatment.

Outcomes

The primary endpoint was the proportion of patients who received the revised hydration regimen (study group) without developing KDIGO-defined acute kidney injury (AKI) stage ≥2 (i.e., serum creatinine ≥2.0-2.9 times baseline) following the first and second chemotherapy treatments. The secondary endpoints were as follows: comparison of the primary endpoint with a historical cohort; the proportion of patients without KDIGO-defined AKI stage ≥1 (i.e., serum creatinine ≥1.5-1.9 times baseline or an increase of ≥0.3 mg/dL) following the first and second chemotherapy treatments; and incidence of hospital admissions and dose reductions or discontinuations of cisplatin due to cisplatin-induced AKI (cis-AKI).

The historical cohort (control group) was selected through data collected from the institutional cancer register and medical records. Individuals in the study and control groups were matched by sex, age, and primary cancer location.

Statistical analysis

The categorical variables are characterized by absolute and relative frequencies. The continuous variables are presented by central tendency and dispersion measures as mean and standard deviation, median and interquartile range. Proportions between groups were assessed through chi-square test. Results were considered statistically significant for p-values less than 0.05. Statistical analyses were performed using R software v4.3.3 (Vienna, Austria: R Core Team).

## Results

Patient characteristics

Recruitment occurred between February 1, 2023, and December 6, 2023, and 105 patients were included, as planned. The demographic and clinical characteristics and creatinine (Cr) baseline values are listed in Table 2.

**Table 2:** Baseline patient characteristics and renal assessment. BSA: body surface area; CrCl: creatinine clearance; ECOG PS: Eastern Cooperative Oncology Group Performance Status

Patient characteristics	Values
Age (min-max)	64 (41-78) years
Male, n (%)	81 (77.1)
ECOG PS, n (%)	
0	47 (44.8)
1	58 (55.2)
Height (median)	1.66 m
Weight (median)	68 kg
BSA (median)	1.757 kg/m^2^
Charlson Comorbidity Index (median)	5
Comorbidities, n (%)	
Arterial hypertension	37 (35.2)
Diabetes	11 (10.5)
Heart failure	7 (6.7)
Previous myocardial infarction	5 (4.8)
Concomitant medication, n (%)	
Renin-angiotensin-aldosterone inhibitors	38 (36.2)
Diuretics	12 (11.4)
Cancer topography, n (%)	
Head and neck	43 (41.0)
Lung	26 (24.8)
Digestive system	21 (20.0)
Urological system	7 (6.7)
Gynecological system	3 (2.9)
Unknown primary site	3 (2.9)
Others	2 (1.9)
Creatinine (median)	0.75 mg/dL
CrCl (median)	91 mL/min

The median age was 64 years and 81 patients (77.1%) were men. The median Charlson Comorbidity Index score was 5. Thirty-seven patients (35.2%) presented arterial hypertension and 38 (36.2%) were receiving renin-angiotensin-aldosterone inhibitors, namely angiotensin-converting enzyme inhibitors (n=24, 22.9%), angiotensin receptor blockers (n=13, 12.4%), and spironolactone (n=1, 1.0%). The most common cancer topographies were head and neck (n=43, 41.0%), lung (n=26, 24.8%), and digestive system (n=21, 20.0%). The median baseline Cr was 0.75 mg/dL and the median baseline CrCl was 91 mL/min.

Renal toxicity

In the study group, the proportion of patients without KDIGO AKI stage ≥2 was 100.0% (n=96) after the first treatment and 97.8% (n=89) after the second treatment. In the control group, the proportion of patients without KDIGO AKI stage ≥2 was 100.0% (n=191) after the first treatment and 98.8% (n=167) after the second treatment. There was no difference between the two groups (p=1.0 for the first treatment and p=0.92 for the second treatment).

The proportion of patients without KDIGO AKI stage ≥1 was 97.9% (n=94) after the first treatment and 93.9% (n=85) after the second treatment. In the control group, the proportion of patients without KDIGO AKI stage ≥1 was 96.9% (n=185) after the first treatment and 94.1% (n=159) after the second treatment. There was no difference between the two groups (p=0.89 for the first treatment and p=1.0 for the second treatment). Cis-AKI was the reason for cisplatin dose reduction in three patients (2.9%) and for treatment discontinuation also in three patients (2.9%). One patient (1.0%) was admitted due to cis-AKI.

Treatment delivery

Treatment delivery details are summarized in Table 3. The median number of chemotherapy cycles was 3 (range: 1-6). Cisplatin was mostly delivered in combination schemes (n=72, 68.6%), more frequently in combination with 5-fluorouracil (n=21, 20.0%), vinorelbine (n=13, 12.4%), and gemcitabine (n=11, 10.5%). Forty-one patients (39.0%) received cisplatin as part of concurrent chemoradiation, as cisplatin monotherapy (n=33, 31.4%), or in combination with 5-fluorouracil (n=8, 7.6%).

**Table 3:** Comprehensive details on treatment delivery.

Treatment characteristic	n (%) (total=105)
Number of cycles	
1	105 (100.0)
2	99 (94.3)
3	79 (75.2)
≥4	52 (49.5)
Chemotherapy scheme	
Cisplatin monotherapy	33 (31.4)
Combined anti-cancer agent	72 (68.6)
5-fluorouracil	21 (20.0)
Vinorelbine	13 (12.4)
Gemcitabine	11 (10.5)
Pemetrexed	8 (7.6)
Docetaxel plus 5-fluorouracil	7 (6.7)
Paclitaxel	5 (4.8)
Others agents	7 (6.7)
Concomitant radiation	41 (39.0)
Curative intent	67 (63.8)
Cisplatin dose reduction	15 (14.3)
Due to renal toxicity	3 (2.9)
Due to other toxicity	10 (9.5)
Due to other reasons, not toxicity	2 (1.9)
Cisplatin discontinuation	32 (30.5)
Due to renal toxicity	3 (2.9)
Due to other toxicity	17 (16.2)
Due to disease progression	4 (3.8)
Due to other reason	8 (7.6)

## Discussion

Hydration is an effective strategy for reducing CIN, apparently by decreasing cisplatin half-life, urinary cisplatin concentrations, and proximal tubule transit time [5,7,11]. Hydration schemes may include intravenous hydration before and/or after cisplatin administration [11]. Two studies demonstrated that oral hydration with an oral rehydration solution (OS-1) or water (at least 1 L per day) may be a safe substitute for post-cisplatin hydration [13,14].

Another CIN prevention strategy may be the use of forced diuresis with mannitol and/or furosemide, which accelerates cisplatin elimination and reduces the concentration of cisplatin in the kidneys [5,11,15]. The optimal dose of mannitol is uncertain due to the heterogeneity of studies, but Li et al. found a better nephroprotective effect when the mannitol dose was ≥25 g [11,15]. Li et al. also demonstrated a better effect of mannitol when cisplatin dose was <75 mg/m^2 ^[15]. On the other hand, Crona et al. concluded that mannitol should be administered when the cisplatin dose is ≥100 mg/m² and/or for patients with preexisting hypertension [5]. The literature suggests that there is no difference between the use of furosemide or mannitol and that there is no benefit in using the combination over a single diuretic [15-18]. It should be noted that furosemide is associated with higher urine output after cisplatin administration and a higher incidence of hyponatremia [18].

Magnesium supplementation is also associated with a nephroprotective effect [3,7,11]. It is suggested that magnesium influences renal transporters, resulting in less cisplatin accumulation in the kidneys [11]. On the other hand, cisplatin results in hypomagnesemia, which potentiates cisplatin accumulation and CIN [3]. Therefore, the literature recommends the systematic addition of magnesium supplementation to the hydration regimen [3,5,11]. A meta-analysis from Hamroun et al. revealed an association between magnesium supplementation and lower risk of AKI [3]. The optimal dose of magnesium supplementation is unclear (studies reported doses between 8 and 20 mEq) [5,11]. In a randomized controlled trial, Suppadungsuk et al. demonstrated that pre-cisplatin administration of 16 mEq magnesium was associated with a lower incidence of cis-AKI [19]. Okamoto et al. carried out a meta-regression analysis that demonstrated a dose-dependent relationship, with higher Mg dosage being more effective in preventing CIN [20].

Urinary magnesium loss is also associated with hypokalemia and hydration regimens often include potassium supplementation (up to 20 mEq) [5,21]. However, the direct role of potassium supplementation in nephroprotection is unknown [5].

In the present study, we demonstrated that a shorter and lower volume intravenous hydration is safe in patients receiving cisplatin-based schemes with a dose >50 mg/m^2^ when compared with a conventional hydration scheme (volume 3000 versus 2500 mL and duration 7 versus 5 hours). Other authors also reported the safety of similar duration and volume hydration schemes (volume: 2000-2500 mL and time: 4-6 h) with cisplatin dose ≥60 mg/m^2^ [22-25]. However, these retrospective studies had some differences from our hydration scheme. Like the present study, Ouchi et al. and Tiseo et al. used normal saline fluids, but Lavolé et al. and Hase et al. have chosen dextrose fluids [22-25]. Both Ouchi et al. and Tiseo et al. used intravenous furosemide 20 mg, while Hase et al. selected 20% mannitol 200 mL [22,24,25]. Tiseo et al. did not use forced diuresis [25]. All patients received magnesium supplementation (8-20 mEq) [22-25]. In addition, replacing post-treatment intravenous hydration with oral hydration seems to be a viable alternative.

This study benefits from a prospective design with a clear, pre-defined hydration protocol, ensuring consistency and minimizing bias. The use of objective renal endpoints (KDIGO criteria) and detailed reporting of patient characteristics enhances the reliability and generalizability of the results. Additionally, the high number of patients and the variety of primary cancer locations increase the study’s reproducibility.

Our study had some limitations. Firstly, in our study, we only analyzed the incidence of cis-AKI given its direct clinical implication in anti-neoplastic treatment, but there are other forms of CIN. Additionally, renal function may also be affected by other factors such as anti-neoplastic agents administered concurrently and tumor lysis. Secondly, the unplanned change in the matching ratio may have affected the statistical power of the study. Lastly, the historical cohort could not be totally comparable because of variances in supportive treatment measures like anti-emesis tactics, and differences in patient characteristics that go beyond those used for matching or confounding factors such as concomitant nephrotoxic drugs.

## Conclusions

This study demonstrates that a shorter-duration, lower-volume intravenous hydration regimen is a safe alternative for patients receiving cisplatin-based chemotherapy at doses >50 mg/m², without increasing the risk of cis-AKI. These findings align with previous studies evaluating similar hydration protocols, reinforcing their feasibility and safety. Additionally, the potential substitution of post-treatment intravenous hydration with oral hydration presents an avenue for further research. Furthermore, we believe that reducing the treatment duration not only allows for better health resource allocation but also improves patient comfort by minimizing hospital stay and treatment burden. Despite some limitations, the prospective design, standardized hydration protocol, and use of objective renal endpoints enhance the reliability and generalizability of our results.
